# Investigation of Bioterrorism-Related Anthrax, United States, 2001: Epidemiologic Findings

**DOI:** 10.3201/eid0810.020353

**Published:** 2002-10

**Authors:** Daniel B. Jernigan, Pratima L. Raghunathan, Beth P. Bell, Ross Brechner, Eddy A. Bresnitz, Jay C. Butler, Marty Cetron, Mitch Cohen, Timothy Doyle, Marc Fischer, Carolyn Greene, Kevin S. Griffith, Jeannette Guarner, James L. Hadler, James A. Hayslett, Richard Meyer, Lyle R. Petersen, Michael Phillips, Robert Pinner, Tanja Popovic, Conrad P. Quinn, Jennita Reefhuis, Dori Reissman, Nancy Rosenstein, Anne Schuchat, Wun-Ju Shieh, Larry Siegal, David L. Swerdlow, Fred C. Tenover, Marc Traeger, John W. Ward, Isaac Weisfuse, Steven Wiersma, Kevin Yeskey, Sherif Zaki, David A. Ashford, Bradley A. Perkins, Steve Ostroff, James Hughes, David Fleming, Jeffrey P. Koplan, Julie L. Gerberding, the National Anthrax Epidemiologic Investigation Team

**Affiliations:** * Centers for Disease Control and Prevention, Atlanta, Georgia, USA; † Maryland Department of Health and Hygiene, Baltimore, Maryland USA; ‡ New Jersey Department of Health and Senior Services, Trenton New Jersey, USA; § Connecticut Department of Public Health, Hartford Connecticut, USA; ¶ District of Columbia Department of Health, Washington D.C., USA; # New York City Department of Health, New York New York, USA; ** Florida Department of Health, Tallahassee, Florida USA

## Abstract

In October 2001, the first inhalational anthrax case in the United States since 1976 was identified in a media company worker in Florida. A national investigation was initiated to identify additional cases and determine possible exposures to Bacillus anthracis. Surveillance was enhanced through health-care facilities, laboratories, and other means to identify cases, which were defined as clinically compatible illness with laboratory-confirmed B. anthracis infection. From October 4 to November 20, 2001, 22 cases of anthrax (11 inhalational, 11 cutaneous) were identified; 5 of the inhalational cases were fatal. Twenty (91%) case-patients were either mail handlers or were exposed to worksites where contaminated mail was processed or received. B. anthracis isolates from four powder-containing envelopes, 17 specimens from patients, and 106 environmental samples were indistinguishable by molecular subtyping. Illness and death occurred not only at targeted worksites, but also along the path of mail and in other settings. Continued vigilance for cases is needed among health-care providers and members of the public health and law enforcement communities.

In the United States, Bacillus anthracis infections have primarily occurred through exposure to infected animals or contaminated animal products such as wool (1). Cases of anthrax have been reported infrequently since the 1970s; the last reported case of inhalational anthrax in the United States occurred in 1976, and the last reported case of cutaneous anthrax occurred in the summer of 2001 (2,3). Outbreaks of inhalational anthrax among humans were linked to occupational exposures at a goat-hair–processing plant in New Hampshire in 1957 and suspected accidental release of B. anthracis aerosols from a bioweapons facility in Sverdlovsk, Russia, in 1979 (4,5). Human cases also have occurred in association with large epidemics of anthrax among animals. Because the bacteria can persist for long periods of time as a spore and can be prepared in a powdered formulation, B. anthracis has been considered a serious biological threat, with potential use as a military or terrorist weapon (6).

After terrorist attacks on the World Trade Center and the Pentagon in 2001, envelopes containing B. anthracis spores were mailed to news media companies and government officials, leading to the first bioterrorism-related cases of anthrax in the United States. We report the combined findings from the epidemiologic and laboratory investigations of these cases, conducted through coordinated efforts of medical and laboratory communities and local, state, and federal public health and law enforcement agencies.

## Methods

Investigators from public health and law enforcement at the federal, state, and local levels collaborated to identify possible cases of anthrax, describe case and exposure characteristics, and prevent further cases through public health interventions. We classified cases as confirmed or suspected on the basis of laboratory and clinical findings (7). A confirmed case of anthrax was defined as clinically compatible illness (cutaneous, inhalational, or gastrointestinal) that was either 1) laboratory confirmed by isolation of B. anthracis from a patient’s clinical specimens, or 2) associated with other laboratory evidence of B. anthracis infection based on at least two supportive tests. A suspected case of anthrax was defined as a clinically compatible illness with no alternative diagnosis and no isolation of B. anthracis, but with either 1) laboratory evidence of B. anthracis by one supportive laboratory test or 2) an epidemiologic link to an environmental B. anthracis exposure.

Laboratory criteria for the case definition of anthrax were 1) isolation of B. anthracis from a clinical specimen from a patient’s affected tissue or site, with confirmation by direct fluorescent-antibody staining and gamma phage lysis (8); or 2) other supportive laboratory tests, including a) evidence of B. anthracis DNA by polymerase chain reaction (PCR) from specimens from a patient’s affected tissue or site, b) demonstration of B. anthracis in a clinical specimen by immunohistochemical staining (IHC), or c) positive serologic testing by an investigational enzyme-linked immunosorbent assay (ELISA) that determined the concentration of serum immunoglobulin G (IgG) to the protective antigen (PA) component of anthrax toxin; sera were considered reactive if antibody was neutralized by competitive inhibition (9,10).

Case finding was initiated by local, state, and federal public health agencies in all 50 U.S. states and through government agencies in other countries. Hospital- and clinic-based surveillance for possible cases of inhalational anthrax in selected regions was done by provider-based reporting and medical record review of patients seen in emergency departments, intensive-care units, and outpatient clinics and in consultation with dermatologists and other medical specialists. Surveillance was also conducted among medical examiners and at affected news media, government, and postal workplaces. Various electronic communication networks of infectious disease physicians, dermatologists, infection control professionals, emergency department physicians, laboratorians, and others were used to increase awareness among practitioners to recognize and report possible cases of anthrax. Case definitions and characteristics, diagnostic and treatment information, and other findings were communicated through the Centers for Disease Control and Prevention (CDC)’s Morbidity and Mortality Weekly Report, Epidemic Information Exchange, and Health Alert Network.

Investigators responded to reports of possible cases from clinicians, law enforcement officials, and the general public. Possible case-patients or exposed persons were interviewed with site-specific data collection forms. Public health laboratories tested clinical specimens, powder-containing envelopes, and environmental samples for the presence of B. anthracis. Demographic data, clinical presentation, exposure risk information, preliminary clinical and environmental laboratory test results, and other findings were collected. Reports of cases meeting the surveillance case definition were forwarded to CDC.

The multistate investigation was conducted by state and local health departments in collaboration with CDC and was coordinated through CDC’s Emergency Operations Center (EOC). The EOC, which used an incident command system structure, was organized into teams of epidemiologists, laboratorians, environmental scientists, communication specialists, and logisticians. EOC teams supported local, state, and federal public health investigators in Florida, New York City, New Jersey, the District of Columbia metropolitan area, and Connecticut. A separate EOC team served as a liaison to state health departments and laboratories. Teams also coordinated interactions with the U.S. Postal Service, Department of Defense, Federal Bureau of Investigation, and other federal agencies and organizations. Intervention teams were initiated to coordinate environmental monitoring and decontamination, postexposure prophylaxis and vaccination, and deployment of National Pharmaceutical Stockpile program assets. Reports of cases and environmental sampling, updates of interventions, and other activities were communicated to the EOC for coordinating the investigation and for communications with federal and state partners, and the media.

Environmental investigations were performed at sites possibly contaminated with B. anthracis spores to assess the presence and extent of contamination and to guide decontamination and environmental remediation. Environmental samples at news media and postal facilities, residences, and other sites were taken by surface sampling with swabs, wipes, HEPA vacuum filtration, and air sampling (11,12). Nasal swab specimens were collected to define the area of exposure to aerosolized B. anthracis and ascertain where a person with inhalational anthrax might have been exposed. Because the sensitivity of nasal swab cultures wanes, attempts were made to obtain cultures within 7 days of exposure. The presence of B. anthracis from nasal swab cultures was not determined by Gram stain or colony characteristics alone but required confirmatory testing by qualified laboratories.

Environmental samples were collected by public health, law enforcement, and contract staff and were tested at laboratories participating with the local, state, and federal investigation efforts. Suspect culture colonies were screened by standard Laboratory Response Network Level A testing procedures for identification of B. anthracis and confirmed by standard Level B procedures, such as direct fluorescent-antibody staining and gamma phage lysis (8,13). Antimicrobial susceptibility patterns were determined for selected B. anthracis isolates by National Committee for Clinical Laboratory Standards (NCCLS) MIC breakpoints for staphylococci (14). NCCLS has not defined either a B. anthracis or staphylococcal interpretive breakpoint for ceftriaxone; thus, breakpoints for gram-negative organisms were used to interpret ceftriaxone results. Isolates of B. anthracis recovered from clinical specimens, environmental samples, and powder-containing envelopes were subtyped to show genetic relationships by multiple-locus variable-number tandem repeat analysis (MLVA) (15). Statistical analysis of epidemiologic data to calculate measures of association was performed by using EpiInfo (CDC, Atlanta, GA) and SAS (SAS Institute, Inc., Cary, NC).

## Results

From October 2 to November 20, 2001, investigators identified 22 cases of bioterrorism-related anthrax; 11 were confirmed as inhalational anthrax and 11 (7 confirmed and 4 suspected) as cutaneous anthrax. The demographic, clinical, and exposure characteristics of each patient are presented in Table 1. In March 2002, an additional case of cutaneous anthrax was reported in a laboratory worker processing environmental samples of B. anthracis in support of the CDC investigation of the fall 2001 bioterrorism-related anthrax attacks (16).

**Table 1:** Demographic, clinical, and exposure characteristics of 22 cases of bioterrorism-related anthrax, United States, 2001

Case no.	Onset date, 2001	Date of anthrax diagnosis by lab testing	State^a^	Age (yrs)	Sex	Race	Occupation	Case status^b^	Anthrax presentation^b^	Outcome	Diagnostic tests
1	9/22	10/19	NY	31	F	W	NY Post employee	Suspect	Cutaneous	Alive	Serum IgG reactive
2	9/25	10/12	NY	38	F	W	NBC anchor assistant	Confirmed	Cutaneous	Alive	Skin biopsy IHC+ / serum IgG reactive
3	9/26	10/18	NJ	39	M	W	USPS machine mechanic	Suspect	Cutaneous	Alive	Serum IgG reactive
4	9/28	10/15	FL	73	M	W, H	AMI mailroom worker	Confirmed	Inhalational	Alive	Pleural biopsy IHC+ / serum IgG reactive
5	9/28	10/18	NJ	45	F	W	USPS mail carrier	Confirmed	Cutaneous	Alive	Skin biopsy IHC+ and PCR+ / serum IgG reac.
6	9/28	10/12	NY	23	F	W	NBC TV news intern	Suspect	Cutaneous	Alive	Serum IgG reactive
7	9/29	10/15	NY	0.6	M	W	Child of ABC employee	Confirmed	Cutaneous	Alive	Skin biopsy IHC+ / blood PCR+
8	9/30	10/4	FL	63	M	W	AMI photo editor	Confirmed	Inhalational	Dead	Cerebrospinal fluid culture +
9	10/1	10/18	NY	27	F	W	CBS anchor assistant	Confirmed	Cutaneous	Alive	Skin biopsy IHC+ / serum IgG reactive
10	10/14	10/19	PA	35	M	W	USPS mail processor	Confirmed	Cutaneous	Alive	Blood culture + / serum IgG reactive
11	10/14	10/28	NJ	56	F	B	USPS mail processor	Confirmed	Inhalational	Alive	Blood PCR+ / pleural fluid cytology IHC+ / serum IgG reactive
12	10/15	10/29	NJ	43	F	A	USPS mail processor	Confirmed	Inhalational	Alive	Pleural fluid IHC+ / bronchial biopsy IHC+ / serum IgG reactive
13	10/16	10/21	VA	56	M	B	USPS mail worker	Confirmed	Inhalational	Alive	Blood culture +
14	10/16	10/23	MD	55	M	B	USPS mail worker	Confirmed	Inhalational	Dead	Blood culture +
15	10/16	10/26	MD	47	M	B	USPS mail worker	Confirmed	Inhalational	Dead	Blood culture +
16	10/16	10/22	MD	56	M	B	USPS mail worker	Confirmed	Inhalational	Alive	Blood culture +
17	10/17	10/29	NJ	51	F	W	Bookkeeper	Confirmed	Cutaneous	Alive	Skin biopsy IHC+ and PCR+ / serum IgG reactive
18	10/19	10/22	NY	34	M	W, H	NY Post mail handler	Suspect	Cutaneous	Alive	Skin biopsy IHC+
19	10/22	10/25	VA	59	M	W	Government mail processor	Confirmed	Inhalational	Alive	Blood culture +
20	10/23	10/28	NY	38	M	W	NY Post employee	Confirmed	Cutaneous	Alive	Skin biopsy culture +
21	10/25	10/30	NY	61	F	A	Hospital supply worker	Confirmed	Inhalational	Dead	Pleural fluid and blood culture +
22	11/14	11/21	CT	94	F	W	Retired at home	Confirmed	Inhalational	Dead	Blood culture +

^a^NY, New York; FL, Florida; NJ, New Jersey; PA, Pennsylvania; VA, Virginia; DC, District of Columbia; MD, Maryland; CT, Connecticut; W, white; B, black; A, Asian; W,H, white with Hispanic ethnicity; NY, New York; NBC, National Broadcasting Company; AMI, American Media Inc.; USPS, United States Postal Service; CBS, Columbia Broadcasting System; PCR, polymerase chain reaction; IHC, immunohistochemical staining; + positive; IgG, immunoglobulin G. ^b^Case status and anthrax presentation are described in the anthrax surveillance case definition in the Methods section.

### Characteristics of Case-Patients

Cases were identified in residents of seven states along the east coast of the United States: Connecticut, one case; Florida, two cases; Maryland, three; New Jersey, five; New York City, eight (includes a case in a New Jersey resident exposed in New York City); Pennsylvania, one; and Virginia, two. The median age of patients was 46 years (range 7 months to 94 years) (Table 2). Patients with inhalational anthrax were older than those with cutaneous disease (56 vs. 35 years, p<0.01). Twelve (55%) patients were male; 15 (68%) were white. Five (23%) case-patients died; deaths occurred only in patients with inhalational anthrax. The case-fatality ratio for inhalational anthrax was 45%. For six cases of inhalational anthrax in postal workers, we were able to estimate the date of first exposure to B. anthracis–positive envelopes processed with high-speed sorters. The mean duration between exposure and onset of symptoms of inhalational anthrax in these patients was 4.5 days (range 4–6).

**Table 2:** Comparison of inhalational and cutaneous bioterrorism-related anthrax cases, United States, 2001

Case characteristic	All cases, n=22 (%)	Inhalational cases n=11, (%)	Cutaneous cases n=11, (%)	p value (inhal. vs. cutan.)
*Median age (range), years^a^*	46 (0.6–94)	56 (43–94)	35 (0.6–51)	<0.01
Male sex (percent)	12 (55)	7 (64)	5 (45)	0.7
Occupation/exposure site^a^				
Mail handler	12 (55)	8 (73)	4 (36)	0.13
Media company employees	6 (27)	1 (9)	5 (45)	
Other	4 (18)	2 (18)	2 (18)	
No./deaths (case-fatality ratio)	5 (23)	5 (45)	0 (0)	0.04
No. of cases following contaminated letters^b^				
September 18 mailing	11 (50)	2 (18)	9 (81)	<0.01
October 9 mailing	8 (36)	7 (64)	1 (9)	

*^a^*Associations suggest that age and occupation varied between inhalational and cutaneous cases; however, it is uncertain if age or occupation were significant independent factors for having a case of anthrax. Wilcoxon two-sample test for nonparametric data was used. All other measurements used two-sided Fisher’s exact test. ^b^Based on documented or presumed paths of contaminated envelopes; excludes three cases-patients who could not be linked to a particular mailing.

All 11 cases of inhalational anthrax met the surveillance definition for a confirmed case; 8 were confirmed by isolation of B. anthracis from a clinical specimen—7 from blood and 1 from cerebrospinal fluid (Table 1). Supportive laboratory tests used to confirm three other cases of inhalational anthrax included IHC or PCR of tissues (pleural biopsy, pleural fluid, or blood) and elevation between acute- and convalescent-phase serum anti-PA IgG by ELISA (9).

Seven (64%) of the 11 cases of cutaneous anthrax met the surveillance definition for a confirmed case; 2 were confirmed by isolation of B. anthracis from a clinical specimen, 1 from blood and 1 from a wound (Table 1). Supportive laboratory tests used in the remaining five confirmed cutaneous cases included IHC or PCR of skin biopsies, PCR of blood, and elevation of serum anti-PA IgG by ELISA. Four cutaneous cases each had only one supportive laboratory test for B. anthracis infection and were classified as suspected: one case had a positive IHC of a skin biopsy, and three had elevated serum anti-PA IgG by ELISA. Among cutaneous anthrax cases, lesions were distributed on the face, arms, or chest; two cases had multiple lesions.

We classified patients into two broad exposure categories on the basis of their primary job duties (Table 2). Twelve (55%) patients (8 with inhalational and 4 with cutaneous disease) were mail handlers, including U.S. Postal Service employees (9 cases), government mail processing staff (1case), and media company mailroom workers (2 cases). Six (27%) patients (one inhalational and five cutaneous cases) were media company employees working at sites where powder-containing mail was received: American Media, Inc. (AMI), one case; Columbia Broadcasting System (CBS), one case; National Broadcasting Company (NBC), two cases; and New York Post, two cases. Four (18%) case-patients (two inhalational and two cutaneous cases) were classified as “other,” including a 7-month-old visitor to the American Broadcasting Company (ABC), a 61-year-old Manhattan hospital supply room worker, a 51-year-old bookkeeper from New Jersey, and a 94-year-old Connecticut resident. For analysis, we excluded case-patients in the “other” category and compared mail handlers with targeted mail recipients. Mail handlers were older (p<0.01) and were associated with inhalational disease (odds ratio [OR] 10; 95% confidence intervals [CI] 0.65 < OR < 530.48; p=0.13). Whether age or occupation were important independent factors in becoming infected is unknown. Of all 22 patients, 20 (91%) either handled mail potentially contaminated with B. anthracis spores or were exposed to worksites where B. anthracis–contaminated mail was processed or received.

### Clinical and Environmental Laboratory Findings

B. anthracis isolates were collected from four powder-containing envelopes, 17 clinical specimens from case-patients, and 106 environmental samples collected along the mail path of the implicated envelopes in Florida, District of Columbia metropolitan area, New Jersey, New York City, and Connecticut. We compared these isolates by MLVA for molecular typing and found that all isolates tested were indistinguishable (17,18). Isolates also had the same antimicrobial susceptibility patterns (18): all isolates tested were susceptible to penicillin (MIC range <0.06 µg/mL–0.12 µg/mL), amoxicillin (MIC <0.06 µg/mL), ciprofloxacin (MIC <0.06 µg/mL), doxycycline (MIC <0.03 µg/mL), chloramphenicol (MIC 4 µg/mL), clindamycin (MIC <0.5 µg/mL), tetracycline (MIC 0.06 µg/mL), rifampin (MIC <0.5 µg/mL), clarithromycin (MIC 0.25 µg/mL), and vancomycin (MIC 1–2 µg/mL). Isolates were borderline susceptible to azithromycin (MIC 2 µg/mL) and intermediate to erythromycin (MIC 1 µg/mL) and ceftriaxone (MIC 16) (19).

### Assessment of Exposures

**Figure 1:**
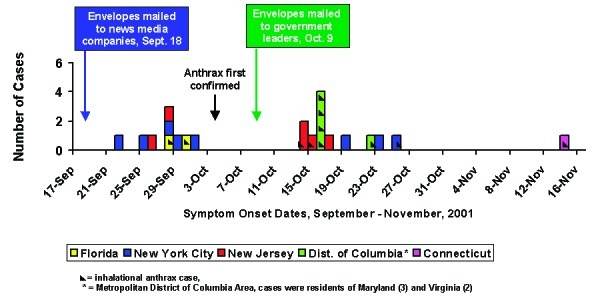
Epidemic curve for 22 cases of bioterrorism-related anthrax, United States, 2001.

Onsets of symptoms occurred from September 22 to November 14, 2001 (Figure 1). Two distinct case clusters were separated in time; no cases occurred during a 13-day period between clusters. One case of inhalational anthrax in a resident of Connecticut occurred 20 days after the second case cluster.

### Envelopes Containing Spores

**Figure 2:**
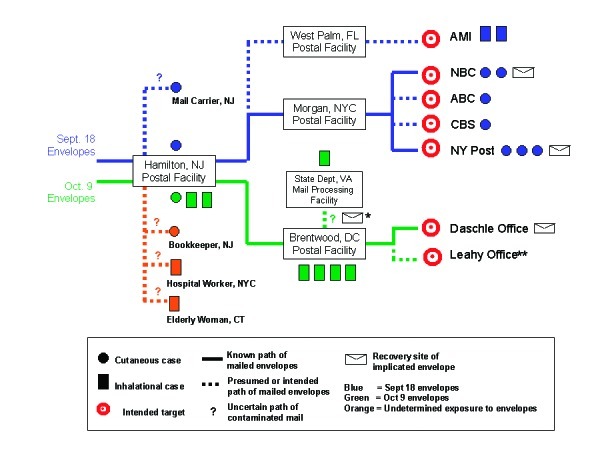
Cases of anthrax associated with mailed paths of implicated envelopes and intended target sites. NY, New York; NBC, National Broadcasting Company; AMI, American Media Inc.; USPS, United States Postal Service; CBS, Columbia Broadcasting System. *Envelope addressed to Senator Leahy, found unopened on November 16, 2001, in a barrel of unopened mail sent to Capitol Hill; **dotted line indicates intended path of envelope addressed to Senator Leahy.

Four B. anthracis–positive powder-containing envelopes were recovered, and the path of the envelopes through the mail was traced (Figure 2). All four envelopes were standard, prestamped U.S. Postal Service issue. Two of the four envelopes, one addressed to NBC news anchor Tom Brokaw and the other to the editor of the New York Post, both in New York City, were mailed in or around Trenton, New Jersey, and were postmarked September 18, 2001. Both these envelopes contained letters with the phrases, “09-11-01…This is next…Take penacilin [sic] now…” (20). The next two envelopes recovered, one addressed to Senator Tom Daschle and one to Senator Patrick Leahy, both in Washington, D.C., were mailed in or around Trenton and were postmarked October 9, 2001. Each envelope contained a letter with statements such as, “09-11-01…You can not stop us. We have this anthrax. You die now. Are you afraid?” No B. anthracis–positive powder-containing envelopes were recovered from other sites in New York City or during investigations in Florida or Connecticut.

The September 18 envelopes were transported through various postal facilities along processing and delivery paths between New Jersey and the intended media company targets in New York City. The implicated envelopes were processed at the U.S. Postal Service Trenton Mail Processing and Distribution Center in Hamilton, New Jersey, and were sent to the Morgan Central Postal Facility in New York City, where they were sorted and delivered. Both these facilities and at least five others in New Jersey affiliated with the Hamilton facility had environmental samples positive for B. anthracis (21,22). Despite environmental evidence of B. anthracis spores at two broadcast media work facilities (ABC, CBS) associated with case-patients, no other B. anthracis–positive mail was recovered. Although no B. anthracis–positive envelopes were recovered in Florida, B. anthracis was isolated from environmental sampling at the AMI building (the worksite of the Florida case-patients) and at least six postal facilities along the path of mail delivered to AMI. The dates of illness onset in AMI media company employees in Florida suggest possible exposure to envelopes mailed in mid-September 2001 (23).

The October 9 envelopes were mailed in or around Trenton, New Jersey, processed at the Hamilton, New Jersey, facility, and transported to the U.S. Postal Service Brentwood Mail Processing and Distribution Center in Washington, D.C. The envelopes were processed with high-speed sorters at both the Hamilton and Brentwood facilities, allowing for the possibility of aerosolized B. anthracis spores. The implicated envelopes and other subsequently contaminated mail were transported to various government mail facilities. One implicated envelope was delivered to the office of Senator Daschle in the Hart Senate Office Building and was opened by office staff on October 15, 2001. Prompt recognition of the potential for anthrax illness from the powder-containing envelope led to rapid initiation of postexposure chemoprophylaxis for exposed office staff. Beginning October 15, nasal swab specimens were collected from 625 persons potentially exposed at the Hart Senate building to the envelope sent to Senator Daschle on October 9; 28 were found to be positive for B. anthracis (24). Environmental sampling showed that sections of the Hart Building and the Brentwood postal facility were heavily contaminated with B. anthracis spores. In addition, at least 25 other government, postal, or mail-receiving facilities affiliated with Brentwood had environmental samples positive for B. anthracis; some of these facilities did not process the implicated envelopes but received other mail from Brentwood. The other implicated envelope postmarked on October 9, 2001, was addressed to Senator Leahy and was recovered unopened on November 16, 2001, in government mail that had been impounded before delivery to Capitol Hill; the exact delivery path of this envelope is unknown (25).

## Case Clusters

The first cluster of nine cases began approximately 4 days after the September 18 envelopes were mailed (Figure 1). All seven cases from New York City and New Jersey in the first case cluster were cutaneous anthrax; all five New York City cases included media company employees or visitors. Both New Jersey cases were in postal employees. The two cases from Florida were both inhalational anthrax and were in media company employees. Overall, eight of the nine persons in the first case cluster were exposed to worksites (postal facilities or media companies) that had environmental samples positive for B. anthracis. One case-patient, a New Jersey mail carrier, had no exposure to any contaminated worksite; exposure to B. anthracis–positive mail, secondarily contaminated at implicated postal facilities (i.e., cross-contaminated mail), is a likely source of infection. The median number of days from the postmark date of September 18, 2001, to onset of illness in the first case cluster was 10 days (range 4–13 days). Onset of illness for all cases in the first cluster occurred before the first culture identification of B. anthracis in the index case of inhalational anthrax in Florida on October 3, 2001 (Figure 1).

The second case cluster began approximately 5 days after the October 9 envelopes were mailed. All five cases from the D.C. metropolitan area were in the second case cluster, all were inhalational anthrax, and all case-patients worked in postal facilities contaminated by the B. anthracis–containing October 9 envelopes. The last two cutaneous cases from New York City whose onsets of illness occurred in the second case cluster (cases numbered 18 and 20 in Table 1) were known to have handled the September 18 New York Post envelope when it was moved in mid-October before its identification. Of the four cases from New Jersey in the second cluster, two were inhalational anthrax in postal employees, one was cutaneous anthrax in a postal worker, and one was cutaneous anthrax in a bookkeeper who worked at a nearby commercial office building; all four case-patients were exposed to worksites that had environmental samples positive for B. anthracis. No definitive B. anthracis exposure was identified for a case of inhalational anthrax in a woman who worked in the supply stockroom of a hospital in Manhattan. Exposure to cross-contaminated mail is a possible source of her infection. The median number of days from the postmark date of October 9, 2001, to onset of illness in the second case cluster was 7 days (range 5–13 days), excluding case-patients with no defined exposure or with exposure to the September 18 envelopes. Thus, the median number of days from mailing of the implicated envelopes to onset of symptoms was an estimated 3 days less for the second cluster; however, there was no statistically significant difference for this comparison.

One case of inhalational anthrax in a 94-year-old female resident of Oxford, Connecticut, had onset of illness on November 14, 2001. No exposure to B. anthracis for this patient could be defined, despite extensive environmental sampling at her home and other sites. Environmental samples at the U.S. Postal Service Wallingford Mail Processing and Distribution Center in Wallingford, Connecticut, were positive for B. anthracis. The Wallingford facility received mail from the contaminated postal facility in Hamilton, New Jersey, and served as the primary source of mail delivered to the patient’s home, suggesting cross-contamination of mail as a possible source of exposure. Postal sorting records indicated that an envelope had been processed in Hamilton on a high-speed sorter 15 seconds after one of the implicated envelopes sent to U.S. senators. That envelope had been delivered to an address 4 miles away from the residence of the Connecticut patient. The envelope was recovered and found to be positive for B. anthracis.

We classified cases on the basis of known or likely exposure to contaminated envelopes, accounting for the location, occupation, and estimated incubation period of the case (Table 2). Eleven cases were associated with the September 18 envelopes (case numbers 1–9, 18, and 20; Table 1). Eight cases were associated with the October 9 envelopes (case numbers 10–16, and 19; Table 1). No certain exposure to any implicated envelopes was found for three cases (case numbers 17, 21, and 22; Table 1). Case number 5, a New Jersey mail carrier, had no exposure to the Hamilton facility or any B. anthracis–positive worksites; however, we classified this case with the September 18 mailing because onset of illness occurred before the October 9 mailing. When we excluded from analysis the three patients who had no definitive exposures, we found that case-patients associated with the September 18 envelopes were more likely to have been exposed at news media facilities than at postal facilities compared with patients associated with the October 9 envelopes (OR undefined, p<0.01). Cases associated with the October 9 envelopes were more likely to be inhalational anthrax than were those associated with the September 18 envelopes (OR 31.5; 95% CI 1.76% to 1,570%; p<0.01). These findings suggest that the October 9 mailing was associated with more severe illness and with development of illness following exposures along the path of the mail.

## Interventions

Antimicrobial postexposure prophylaxis was recommended for persons at risk for inhalational anthrax given 1) the presence of an inhalational case at a facility (e.g., AMI in Florida), 2) environmental specimens positive for B. anthracis in facilities along the path of a contaminated letter where aerosolization might have occurred (e.g., postal facilities in New York City, New Jersey, Connecticut, District of Columbia, and Virginia), and 3) exposure to an air space known to be contaminated with aerosolized B. anthracis from an opened letter (e.g., Senate office buildings in the District of Columbia) (26,27). An estimated 32,000 persons initiated antimicrobial prophylaxis; however, completion of a 60-day course of antimicrobial prophylaxis was recommended for approximately 10,300 persons who met the factors listed above (26–28). Because some persons requested additional precautions, especially those exposed to high levels of anthrax spores, more antibiotics—alone or with vaccine—were offered to other persons in the same cohort (29). No additional cases of anthrax have been reported in persons at sites where B. anthracis exposures were suspected and where exposed persons initiated antimicrobial prophylaxis. Additional description of antimicrobial postexposure prophylaxis is presented elsewhere (30–32).

## Discussion

We identified 22 cases of anthrax that occurred after envelopes containing B. anthracis–positive powder were mailed to persons in news media and government. Inhalational and cutaneous disease followed exposure to B. anthracis spores; five people died. These cases represent the first reported bioterrorism-related outbreak of anthrax. The investigation of these cases reveals important findings for detecting and preventing infections from bioterrorist attacks.

We tested B. anthracis isolates from patients, powder-containing envelopes, and environmental samples from news media, government, and postal processing worksites and found all tested isolates to be indistinguishable by molecular typing methods. Similar U.S. postal service-issue envelopes containing powder preparations of these B. anthracis spores were mailed from the Trenton, New Jersey, area on at least two dates. Although isolates, envelopes, and originating postal paths were similar, characteristics of cases differed by date of mailing and geographic region.

Patients in the cluster that occurred after the September 18 mailing were more likely to have cutaneous disease and to have been exposed at news media facilities rather than at postal facilities. Case-patients in the cluster that occurred after the October 9 mailing were more likely to have inhalational disease and to have been exposed at postal facilities along the path of envelopes sent to U.S. senators. Postal workers exposed to B. anthracis from the October mailings had predominantly inhalational disease. The case-fatality ratio for all cases of inhalational anthrax was 45%, a ratio lower than previously reported (33); the estimated incubation period of 4.5 days for inhalational cases was consistent with previously reported findings (1).

The fulminant systemic illness associated with the October mailing to U.S. senators differed greatly from the less severe cutaneous cases in media company employees in New York City, suggesting that substantial illness and death likely might have occurred among senate office staff after implicated envelopes were opened. Exposure to B. anthracis spores from processing unopened envelopes at the Hamilton and Brentwood postal facilities went unrecognized until after the implicated envelope was opened at the Hart Senate Office Building. Administration of postexposure chemoprophylaxis likely prevented further cases in postal workers and almost certainly averted disease in senate staff. Estimates derived from mathematical models support this conclusion (34). Our findings suggest that prompt use of antimicrobial prophylaxis following suspected bioterrorist attacks can prevent disease.

Differences in the consistency of B. anthracis powders between the September and October mailings have been reported by the Federal Bureau of Investigation and may account for the preponderance of inhalational cases in the second cluster (35,36). The later mailings may have intentionally contained a smaller particle-sized powder to produce greater harm. Media company employees had less severe disease than did the postal workers along the path of envelopes sent to senators.

Our findings indicate that the clinical and epidemiologic presentations of a bioterrorist attack depend on the population targeted, the characteristics of the agent, and the mode of transmission. With naturally occurring outbreaks of infection, early cases identified often provide clues to the mode and source of exposure. For bioterrorism-related disease, characteristics of initial cases may be misleading if terrorists vary the mode and source of exposure. Further understanding is needed of the role of different B. anthracis powder formulations in the mode of exposure and illness characteristics of persons exposed.

Cases of anthrax occurred in persons near those targeted for infection and also in those along the mail path of spore-containing envelopes. After the mailing of the September 18 envelopes, cases of cutaneous anthrax occurred, but were initially unrecognized, in workers at the postal processing center in New Jersey where the implicated envelopes originated. After the mailing of the October 9 envelopes, inhalational disease was identified in workers at postal facilities in the District of Columbia and New Jersey. Investigators did not anticipate the exposures and fulminant disease in those exposed to aerosols of B. anthracis spores from unopened envelopes along the path of the mail. No prior experience with mailed B. anthracis–positive, powder-containing envelopes is described in published reports; previous descriptions of aerosolized B. anthracis spores indicated that risk for re-aerosolization or resuspension of spores was low (37). Previous preventive strategies for presumed B. anthracis exposures now appear inadequate in light of recent findings. Before this incident, antimicrobial prophylaxis was recommended only for direct exposures to the envelopes, and limited decontamination was suggested only for the immediate site of envelope opening (38). Cutaneous and inhalational disease in postal workers in our investigation clearly shows that sealed, B. anthracis–positive, powder-containing envelopes can be a source of infection, presumably via the airborne route, for persons processing contaminated mail in postal facilities. Airborne transmission at the Brentwood and Hamilton facilities may have been facilitated by the use of high-speed sorters, as well as air-blowers used for routine cleaning (12). Any future investigations of bioterrorism-related anthrax should evaluate persons potentially exposed along the path of the delivery vehicle as well as those targeted by the attack.

We found most cases of anthrax to be epidemiologically linked to sites contaminated by implicated envelopes; however, not all cases had direct exposures to targeted worksites, implicated envelopes, or mail-processing facilities along the mail path. Two cutaneous anthrax patients, a mail carrier and a bookkeeper in New Jersey, were not exposed to contaminated postal facilities or media companies. Only one of many environmental samples of surfaces at the bookkeeper’s office, where mail was received, was positive for B. anthracis. Cross-contaminated mail may be a likely exposure source for anthrax for both these cases.

The possibility of B. anthracis exposure from envelopes secondarily contaminated from implicated postal facilities greatly extended the group of potentially exposed persons in our investigation. Experience with anthrax related to agricultural or industrial sources indicated that direct exposure to animals, animal products, and wool-processing facilities accounted for most reported cases (1,3,4,39). Contamination of the environment in animal and wool-processing facilities has been shown, and occasional cases due to secondarily contaminated items have been reported as a possible source of anthrax (1).

For our investigation, contamination found at postal processing facilities off the direct mail path of implicated envelopes indicates that cross-contamination of mail occurred; however, enhanced surveillance for anthrax cases in multiple regions has not identified additional cases. Two patients with inhalational anthrax, a hospital worker in New York City and a retired woman in Connecticut, had no exposure to media or government worksites, implicated postal facilities, or possible sources of naturally occurring anthrax (40). Neither patient had evidence of B. anthracis contamination at her home (or workplace for the New York City case), yet both were infected with B. anthracis isolates indistinguishable from the outbreak strain. Postal processing facilities in New York City and Wallingford, Connecticut, were contaminated with B. anthracis, suggesting cross-contaminated mail as a possible source of B. anthracis exposure for both cases.

From our investigation, B. anthracis–positive powder appears capable of contaminating other mail during processing, leading to exposure and subsequent development of cutaneous and possibly inhalational anthrax. The risk from cross-contaminated mail appears to be extremely low; 85 million pieces of mail were processed at facilities in New Jersey and District of Columbia after the October 9 envelopes, and no additional anthrax cases were detected through stimulated enhanced hospital-based surveillance of 10.5 million people in metropolitan areas around those postal facilities (41). Although the risk for B. anthracis infection from cross-contaminated mail may be low, investigations of future bioterrorist attacks with B. anthracis–positive powders should consider the potential role of secondarily contaminated items in transmission of disease. An attack using a greater number of spore-containing envelopes would likely lead to many more cases due to cross-contaminated mail (42).

Throughout the investigation, various reporting mechanisms were used to enhance detection of cases, including prospective syndromic surveillance in emergency departments and intensive-care units, laboratory-based surveillance, networks of clinicians such as dermatologists, and worksite absenteeism monitoring. In general, most cases of anthrax were detected through reports from clinical laboratorians and clinicians and from patient self-reporting. The role of the news media in increasing patient, clinician, and laboratorian awareness of anthrax was likely an important factor in stimulating case detection and reporting. Health departments sent alerts to health-care providers and provided training seminars for clinicians to improve case detection. Before the bioterrorism-related anthrax cases in 2001, clinician recognition of clinical findings suggestive of cutaneous or inhalational anthrax is presumed to have been very low (43,44). For our investigation, cases in the first cluster associated with the September 18 mailing went unrecognized until B. anthracis was identified in a culture of cerebrospinal fluid from the index case in Florida, underscoring the critical role of the laboratory in initiating the investigation.

These first unrecognized cutaneous cases demonstrate the potential difficulties in detecting cases from a covert bioterrorism agent release. Once the possibility of anthrax exposures at media companies was recognized, along with subsequent environmental work site samples positive for B. anthracis, cases of cutaneous anthrax were more readily detected and reported. During the investigation, rapid dissemination of clinical findings through broadcast e-mail and fax alerts to hospitals and providers, public health reports, and networks of clinical, laboratory, and public health officials provided important tools to frontline clinicians to improve recognition of anthrax. Enhancing the knowledge and skills of clinicians and laboratorians for diagnosing bioterrorism-related infections and improving collaborations between clinicians and public health practitioners will set the stage for better detection of cases associated with any future acts of bioterrorism.

Our investigation had several limitations. The detection of anthrax cases involved numerous local, state, and federal public health and law enforcement officials. Because of the widely distributed activities of various investigators and the need to act quickly in identifying potential exposure sources, data collection instruments were not uniform. Collation of information across sites was limited to a select set of demographic, exposure, and risk factor data elements. The wide use of postexposure prophylaxis, along with difficulty in obtaining detailed information about potentially exposed persons, prevented general estimates of anthrax attack rates for many sites. Surveillance case definitions required laboratory confirmation of disease or of environmental exposure and thus may have missed cases of disease that were treated empirically without appropriate cultures (e.g., illness empirically treated as infected spider bites, which was actually cutaneous anthrax). Environmental sampling of potentially contaminated facilities used different testing methods; because less sensitive testing methods were used, certain sites may have underrepresented the degree of contamination. Throughout the investigation, there was a continuing need to refine study methods and redetermine intervention recommendations, since prior experience with bioterrorism-related anthrax was lacking. Finally, because the public health investigation was also a criminal investigation, information that may have contributed epidemiologic information may not have been available to many public health investigators because it was protected for use in prosecution.

The attacks initiated response activities in all states across the United States and in other countries and required considerable resources to support investigative efforts at the local, state, and federal levels. The perpetrator has not been apprehended, and new cases can still occur. Continued collaboration with law enforcement officials is required, and clinicians, laboratorians, public health officials, and the general public should remain alert for patient symptoms or findings that might indicate additional cases of bioterrorism-related anthrax.
