# Correction, Vol. 8, No. 10

**DOI:** 10.3201/eid0901.020354

**Published:** 2003-01

In “Investigation of Bioterrorism-Related Anthrax, United States, 2001: Epidemiologic Findings” by Daniel B. Jernigan et al., errors occurred in the listing of the members of the Anthrax Epidemiologic Investigation Team on page 1019, http://www.cdc.gov/ncidod/EID/vol8no10/02-0353.htm

Additional members of the National Anthrax Epidemiologic Investigation Team are: Francisco Alvarado-Ramy, MacKenzie Andre, MaryKate Appicelli, Mick Ballesteros, Mark Beatty, Omotayo Bolu, Louise Causer, Soju Chang, Ilin Chuang, John Crump, Marvin DeBerry, Rachel Gorwitz, Michelle Goveia, Thomas Handzel, Josh Harney, Dan Hewett, Vincent Hsu, Young Hur, Marialena Jefferds, Joshua Jones, Kathleen Julian, Richard Kanwal, Jane Kelly, Dennis Kim, Judy Kruger, Richard Leman, Steve Lenhart, Jill Levine, Naile Malakmadze, Els Mathieu, Rob McCleery, Shawn McMahon, Manoj Menon, Kelly Moore, Jill Morris, James Andy Mullins, Melanie Myers, Timothy Naimi, Lori Newman, Chima John Ohuabunwao, Michael O'Reilly, Lisa Pealer, Chris Piacitelli, Joe Posid, John Redd, Mary Reynolds, Julia Rhodes, Louie Rosencrans, Lisa Roth, Denise Roth-Allen, Sharon Roy, Taraz Samandari, Dejana Selenic- Stanacev, Jina Shah, Tanya Sharp, Allison Stock, Lauralynn Taylor, Pauline Terebuh, Christopher Thomas, Beth Tohill, Barna Tugwell, Angela Weber, Dana White, Sara Whitehead, Wally Wilhoite, Leigh Winston, Brad Winterton, Katharine Witgert, William Wong, Susie Wootton, and Weigong Zhou

We regret any confusion these errors may have caused.

